# A retrospective and prospective study to establish a preoperative difficulty predicting model for video-assisted thoracoscopic lobectomy and mediastinal lymph node dissection

**DOI:** 10.1186/s12893-022-01566-3

**Published:** 2022-04-08

**Authors:** Zixiao Wang, Yuhang Wang, Daqiang Sun

**Affiliations:** 1 Tianjin Medical University, Heping, Tianjin, 300070 People’s Republic of China; 2 Department of Thoracic Surgery, Tianjin Chest Hospital, Jinnan, Tianjin, 300222 People’s Republic of China; 3 Department of Thoracic Surgery, Tianjin Hospital of ITCWM Nankai Hospital, No. 6 Changjiang Road, Nankai, Tianjin, 300100 People’s Republic of China

**Keywords:** Surgical difficulty, Thoracoscopic lobectomy, Mediastinal lymph node dissection, Predicting model, Nomogram

## Abstract

**Background:**

In previous studies, the difficulty of surgery has rarely been used as a research object. Our study aimed to develop a predictive model to enable preoperative prediction of the technical difficulty of video-assisted thoracoscopic lobectomy and mediastinal lymph node dissection using retrospective data and to validate our findings prospectively.

**Methods:**

Collected data according to the designed data table and took the operation time as the outcome variable. A nomogram to predict the difficulty of surgery was established through Lasso logistic regression. The prospective datasets were analyzed and the outcome was the operation time.

**Results:**

This retrospective study enrolled 351 patients and 85 patients were included in the prospective datasets. The variables in the retrospective research were selected by Lasso logistic regression (only used for modeling and not screening), and four significantly related influencing factors were obtained: FEV1/FVC (forced expiratory volume in the first second/forced vital capacity) (*p* < 0.001, OR, odds ratio = 0.89, 95% CI, confidence interval = 0.84–0.94), FEV1/pred FEV1 (forced expiratory volume in the first second/forced expiratory volume in the first second in predicted) (*p* = 0.076, OR = 0.98, 95% CI = 0.95–1.00), history of lung disease (*p* = 0.027, OR = 4.00, 95% CI = 1.27–15.64), and mediastinal lymph node enlargement or calcification (*p* < 0.001, OR = 9.78, 95% CI = 5.10–19.69). We used ROC (receiver operating characteristic) curves to evaluate the model. The training set AUC (area under curve) value was 0.877, the test set’s AUC was 0.789, and the model had a good calibration curve. In a prospective study, the data obtained in the research cohort were brought into the model again for verification, and the AUC value was 0.772.

**Conclusion:**

Our retrospective study identified four preoperative variables that are correlated with a longer surgical time and can be presumed to reflect more difficult surgical procedures. Our prospective study verified that the variables in the prediction model (including prior lung disease, FEV1/pred FEV1, FEV1/FVC, mediastinal lymph node enlargement or calcification) were related to the difficulty.

## Background

Lung cancer has a high incidence and mortality [1]. According to the 2020 epidemiological study statistics, the lung cancer incidence is in second place among cancers, and its mortality rate among cancers is number one worldwide [1, 2]. The NCCN (National Comprehensive Cancer Network) guidelines recommend that patients with lung cancer without obvious surgical or anatomical contraindications should consider minimally invasive tumor resection [3]. Recently, studies have reported that VATS (video-assisted thoracoscopic surgery) lobectomy accounts for a high proportion of thoracic surgeries [4]. Although VATS lobectomy is common, it has a certain complexity and high surgical risk. The difficulty of operations is a problem of great concern to patients and surgeons. At present, there is no clear approach to conducting a preoperative evaluation to predict the difficulty of a type of surgery. In the past, the difficulty of surgery was generally based on subjective evaluation by the surgeon, and few studies applied objective indicators. Therefore, it is necessary to establish a model for evaluating the difficulty of surgery. This study aimed to establish a preliminary evaluation model and used retrospective data to predict the difficulty of VATS lobectomy. We then validated our findings in a prospective study.

## Methods

### Retrospective data

#### Study population

The retrospective data included patients who underwent VATS lobectomy and mediastinal lymph node dissection at our center from 2016.1.1 to 2020.3.31, which were all performed by the same surgeon (an experienced chief thoracic surgeon). The size of the main lesion was less than 3 cm, the surgical site was only a single lobe, intraoperative frozen pathology identified lung cancer, and mediastinal lymph node dissection was performed. There were 2 incisions (i.e., an endoscopic observation site and an operation site) for all patients. Patients who were diagnosed with lung cancer before an operation or only underwent lobectomy without lymph node dissection were excluded. Those with lesions not limited to a single lobe (clearly defined as lung cancer lesions) were excluded. Those who underwent multilobe or sublobe surgery or underwent neoadjuvant therapy before the operation were also excluded. All the patients received written informed consent prior to the surgery. The study was approved by Ethics Committee of Department of Thoracic Surgery, Tianjin Chest Hospital.

#### Data collection and statistical analysis

**Fig. 1:**
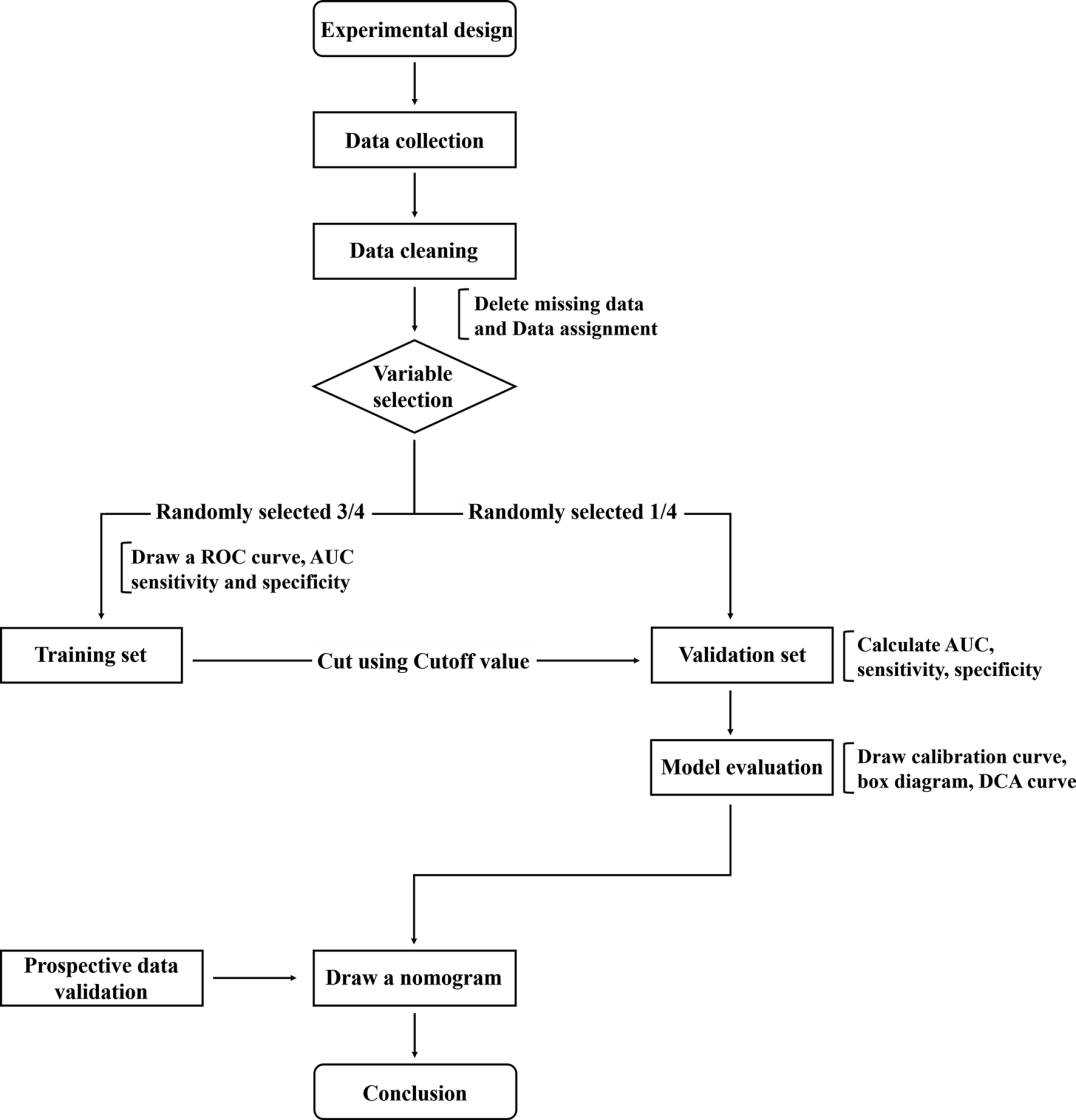
Flow chart of the research

We collected the preoperative information and possible influencing factors of the patients, including the patients’ gender, age, height, weight, liver and kidney function, routine blood tests, coagulation six items, and smoking history. We also collected data relevant to the surgical treatment, including a history of lung diseases (such as prior lung surgery, tuberculosis and obstructive pneumonia), the presence of coronary heart disease (some of the patients were first diagnosed with coronary heart disease and then pulmonary nodules were found incidentally), lung function assessments, and whether mediastinal lymph node enlargement or calcification was observed on chest CT. The operation time was selected as the outcome variable and used to represent the difficulty of the operation. R, version 4.0.4 was used to perform all of the data analyses. We used Lasso logistic regression analysis to build the model. We chose the variables that were retained when *λ* = 1•se (standard eorror) as the variables used to build the Logistics regression model. The model was evaluated with ROC (Receiver Operating Characteristic) curves, calibration curves, and DCA (Decision Curve Analysis) curves, and finally a nomogram was constructed [5, 6]. The process is shown in Fig. 1.

### Prospective data

#### Study population and data collection

*Data collection*: Patients met the admission criteria (same to the retrospective study’s criteria) for VATS lobectomy and mediastinal lymph node dissection in our center 2021.4.1 to 2021.10.31 were included. We collected preoperative information from all patients following the data collection form designed for the retrospective study. We incorporated the data into the model constructed from the retrospective data.

## Results

### The results of the retrospective data

**Fig. 2:**
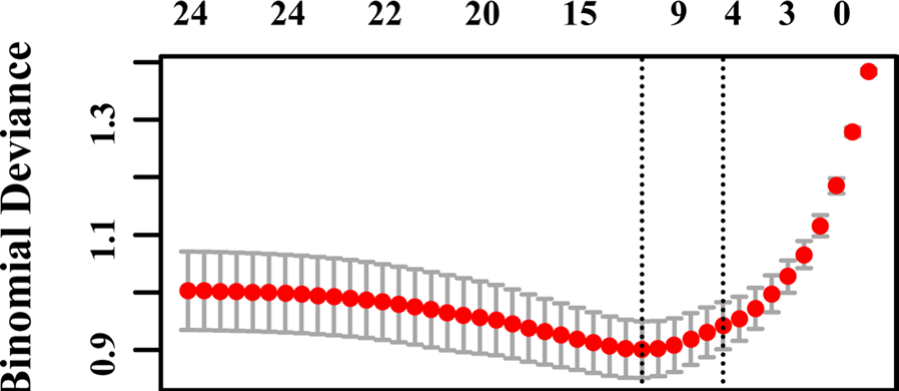
Lasso regression screened four significant preoperative variables

**Fig. 3:**
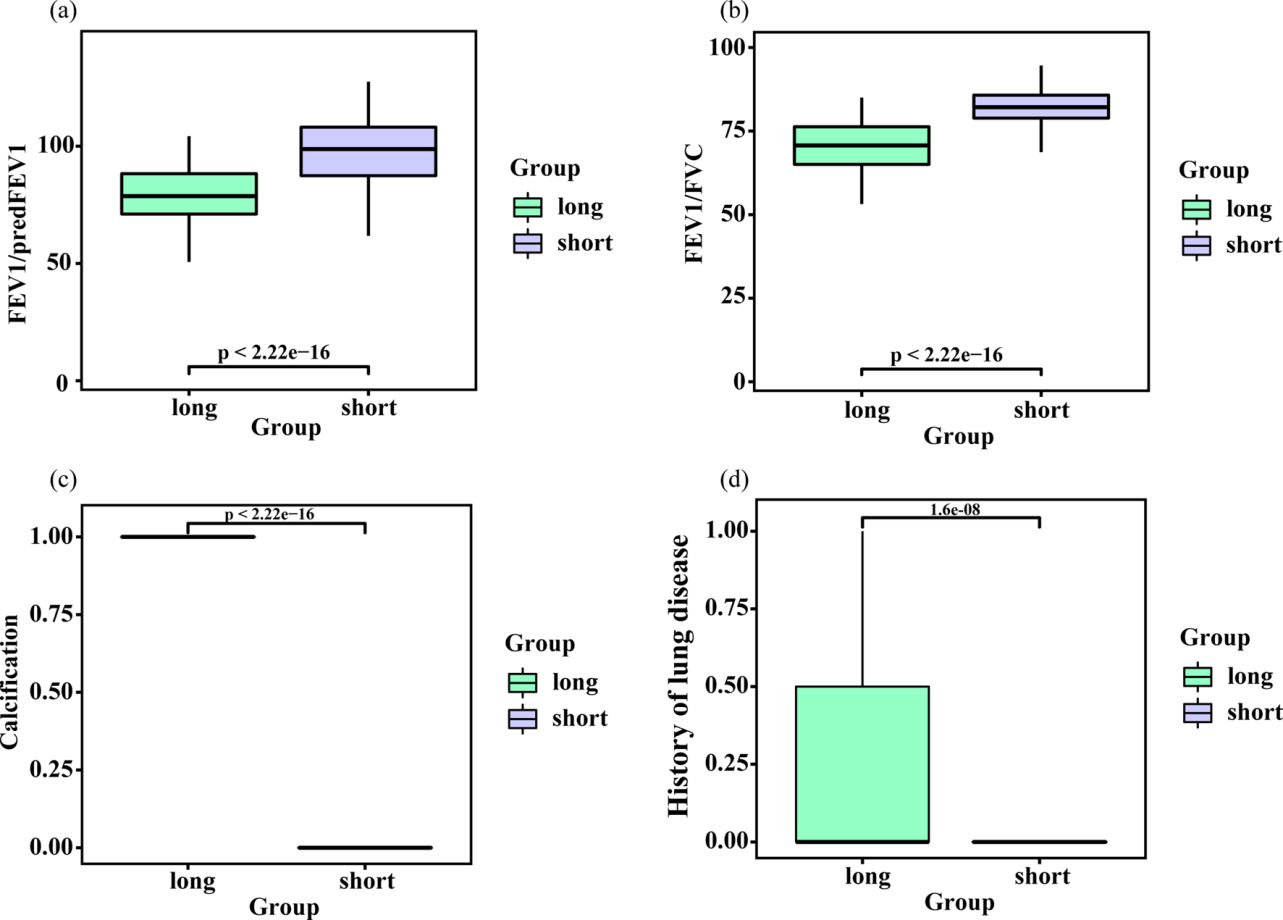
Box plots showed four significantly different variables including FEV1/pred FEV1 (**a**), FEV1/FVC (**b**), mediastinal lymph node enlargement or calcification (**c**) and prior lung disease (**d**) for two groups in a training set

**Fig. 4:**
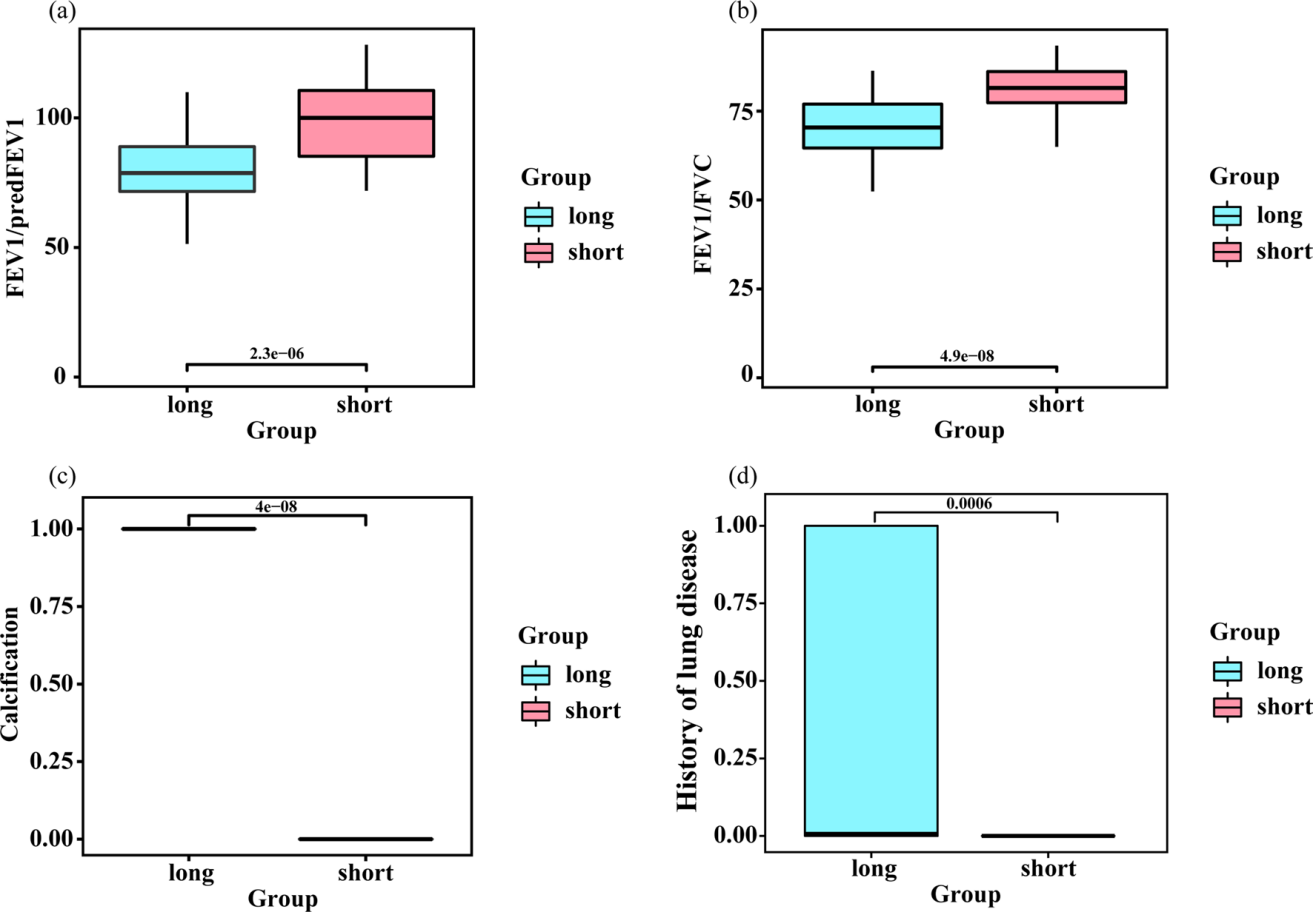
Box plot shows four significantly different variables including FEV1/pred FEV1 (**a**), FEV1/FVC (**b**), mediastinal lymph node enlargement or calcification (**c**) and prior lung disease (**d**) for two groups in a test set

**Fig. 5:**
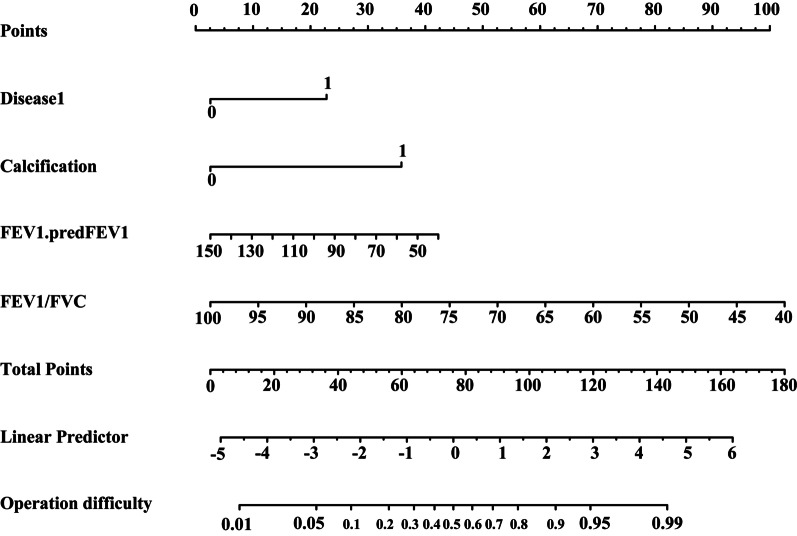
A nomogram was established by four preoperative variables

There were 437 patients who met the criteria. After removing some samples with missing data, a total of 351 cases were finally included and 25 variables were evaluated (according to the sample accounts: variable > 10:1). The 25 variables are as follows: age, gender, prior lung disease, coronary heart disease, smoking, lobe, mediastinal lymph node enlargement or calcification, height, weight, FVC/ pred FVC, FEV1/predFEV1, FEV1/FVC, DLCO.SB, white blood cell, absolute neutrophil count, red blood cell, hemoglobin, prothrombin time, activated partial thromboplastin time, thrombin time, D. dimer, albumin, creatinine, fissure, and neutrophilic granulocyte percent. A total of 351 patients were randomly divided into a training set and a test set at a ratio of 3 to 1. Their detailed baseline information is shown in Tables 1 and 2. All patients in the group were divided into two groups according to the median operation time. The 25 variables were screened by Lasso regression, and the variables that had a significant impact on the operation time were as follows: (1) Whether they had a history of lung disease (history of lung surgery) (2) Whether they had mediastinal lymphadenopathy or calcification. (3) FEV1/FVC (forced expiratory volume in the first second/forced vital capacity) on the lung function test. (4) FEV1 as a percentage of the predicted value on the lung function test. Although the remaining variables differed among the patients, the results were not statistically significant (Fig. 2). The differences in the operation time of the training set and the validation set between the two groups for the related variables are shown in Figs. 3 and 4. The above variables were found to be independent risk factors for VATS lobectomy and were included in multifactor logistic regression analysis. We only used multifactor logistics regression to establish the model and did not use it to further screen the relevant variables after screening by Lasso regression. Finally, a nomogram was established to predict the difficulty of the surgery (Fig. 5).

**Table 1:** Baseline data from training set

Level		Short (n = 132)	Long (n = 137)	p
Age		59.82 ± 8.72	62.41 ± 8.4	0.014
Gender (%)	0	70 (53.0)	46 (33.6)	0.002
	1	62 (47.0)	91 (66.4)	
Disease1 (%)	0	128(97.0)	109 (79.6)	< 0.001
	1	4 (3.0)	28 (20.4)	
Disease2 (%)	0	105 (79.5)	119 (86.9)	0.149
	1	27 (20.5)	18 (13.1)	
Smoking (%)	0	87 (65.9)	58 (42.3)	< 0.001
	1	45 (34.1)	79 (57.7)	
Lobe (%)	1	27 (20.5)	42 (30.7)	0.004
	2	31 (23.5)	12 (8.8)	
	3	37 (28.0)	53 (38.7)	
	4	8 (6.1)	7 (5.1)	
	5	29 (22.0)	23 (16.8)	
Calcification (%)	0	100 (75.8)	38 (27.7)	< 0.001
	1	32 (24.2)	99 (72.3)	
Height		164.86 ± 7.98	167.12 ± 8.38	0.024
Weight		67.04 ± 10.89	69.82 ± 12.23	0.050
FVC/ pred FVC		99.25 ± 14.67	93.28 ± 13.53	0.001
FEV1/predFEV1		96.98 ± 14.52	83.42 ± 15.41	< 0.001
FEV1/FVC		81.67 ± 6.97	72.89 ± 8.69	< 0.001
DLCO.SB		84.91 ± 12.90	83.75 ± 14.33	0.487
White blood cell		6.08 ± 1.86	6.48 ± 1.80	0.075
Absolute neutrophil count		3.75 ± 1.51	4.10 ± 1.56	0.059
Red blood cell		4.47 ± 0.49	4.86 ± 3.99	0.261
Hemoglobin		135.20 ± 14.95	139.11 ± 15.46	0.036
Prothrombin time		12.93 ± 0.61	13.01 ± 0.62	0.269
Activated partial thromboplastin time		35.70 ± 3.37	36.47 ± 4.33	0.107
Thrombin time		16.84 ± 1.05	16.69 ± 1.13	0.275
D. dimer		0.47 ± 0.51	0.48 ± 0.71	0.847
Albumin		43.67 ± 4.10	43.75 ± 4.40	0.887
Creatinine		69.21 ± 12.33	73.36 ± 17.30	0.025

**Table 2:** Baseline data from validation set

Level		Short (n = 42)	Long (n = 40)	p
Age		62.48 ± 8.70	63.40 ± 9.07	0.639
Gender (%)	0	15 (35.7)	17 (42.5)	0.687
	1	27 (64.3)	23 (57.5)	
Disease1 (%)	0	41 (97.6)	30 (75.0)	0.007
	1	1 (2.4)	10 (25.0)	
Disease2 (%)	0	37 (88.1)	36 (90.0)	1.000
	1	5 (11.9)	4 (10.0)	
Smoking (%)	0	25 (59.5)	20 (50.0)	0.519
	1	17 (40.5)	20 (50.0)	
Lobe (%)	1	9 (21.4)	5 (12.5)	0.047
	2	6 (14.3)	1 (2.5)	
	3	14 (33.3)	24 (60.0)	
	4	1 (2.4)	3 (7.5)	
	5	12 (28.6)	7 (17.5)	
Calcification (%)	0	37 (88.1)	8 (20.0)	< 0.001
	1	5 (11.9)	32 (80.0)	
Height		167.57 ± 7.52	166.52 ± 10.04	0.594
Weight		70.60 ± 8.45	72.47 ± 12.01	0.413
FVC/Pred FVC		97.72 ± 13.52	95.35 ± 14.40	0.445
FEV1/predFEV1		95.85 ± 15.49	87.05 ± 16.87	0.016
FEV1/FVC		81.06 ± 7.14	73.65 ± 9.94	< 0.001
DLCO.SB		87.34 (15.08	86.52 ± 11.24	0.783
White blood cell		5.94 ± 1.35	6.93 ± 2.17	0.015
Absolute neutrophil count		3.80 ± 1.10	4.44 ± 2.16	0.093
Red blood cell		4.47 ± 0.53	4.48 ± 0.41	0.915
Hemoglobin		136.93 ± 14.32	138.38 ± 13.22	0.636
Prothrombin time		13.10 ± 0.95	12.96 ± 0.60	0.405
Activated partial thromboplastin time		36.82 ± 3.05	35.93 ± 3.17	0.200
Thrombin time		16.88 ± 1.18	16.91 ± 1.18	0.897
D. dimer		0.37 ± 0.37	0.53 ± 0.99	0.337
Albumin		42.95 ± 4.85	44.28 ± 3.58	0.165
Creatinine		71.52 ± 12.22	71.53 ± 15.70	1.000

^*^Gender 1: man 2. women

Lobe: 1. left upper 2. left lower 3. right middle 4. right lower 5. right upper

Disease 1: prior lung disease 0 none 1 exist

Disease2: coronary heart disease 0 none 1 exist

Calcification: 0 none 1 exist

Smoking: 0 none 1 exist

DLCO.SB: Percentage of carbon monoxide diffusing capacity to predicted capacity

**Fig. 6:**
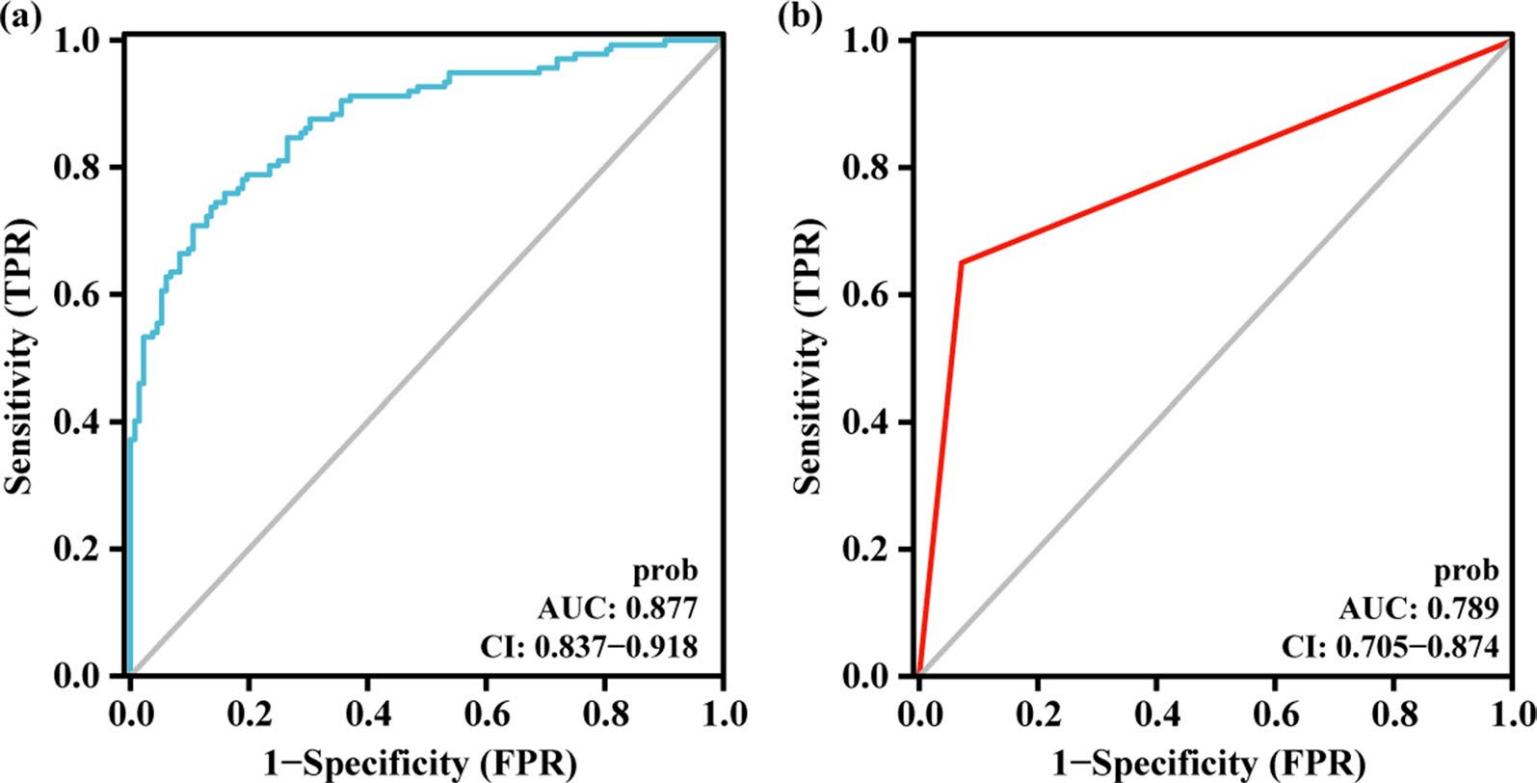
The ROC curves showed that the training set AUC value was 0.877 and the test set’s AUC was 0.789

**Fig. 7:**
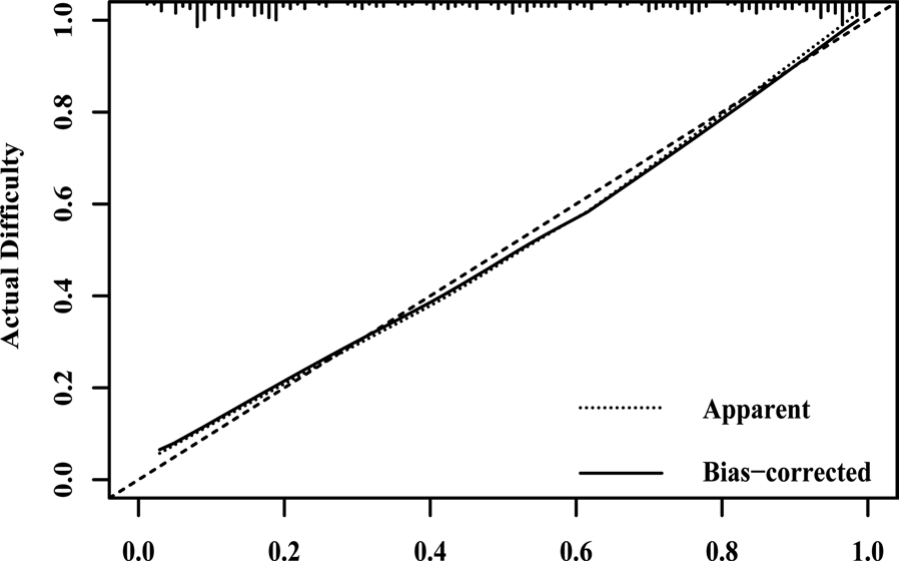
The calibration curve shows that the results of the model in predicting the difficulty are consistent with the practice

**Fig. 8:**
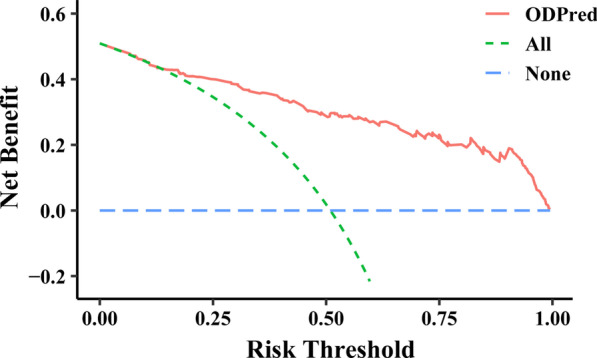
The DCA curves demonstrated that the net benefits obtained from the model were considerable. *ODPred* operation difficulty predicting model

*Evaluation of the prediction nomogram results:

After calculating the ROC (receiver operating characteristic) curve, the AUC (area under curve) value in the training set was 0.877 (Fig. 6a). The cut-off value was calculated at this time and applied to the verification set, and its ROC curve was calculated and the AUC was 0.789 (Fig. 6b). This model thus has a stable discrimination ability. In addition, a calibration curve was drawn. The calibration curve showed that the accuracy of the nomogram in predicting difficult operations (long surgery time) was consistent with the actual situation (Fig. 7). In addition, we produced a clinical decision curve, and its results also showed that the model had good discriminative ability (Fig. 8).

### The results of the prospective data

The final number of patients who underwent VATS lobectomy and mediastinal lymph node dissection was 85. We followed the scheme shown in the flowchart and fitted the prediction results for the prospective data (AUC = 0.772), which confirmed the accuracy of the model established in our retrospective analysis and validated our research (Table 3).

**Table 3:** Comparison between model prediction and practice in prospective research

		Actual		Total
		Long	Short	
ODPred	Long	24	6	30
	Short	12	43	55
Total		36	49	
Sensitivity		0.667		
Specificity			0.878	

*ODPred* operation difficulty predicting model

## Discussion

### About the results

In recent years, with the application of low-dose chest CT in the early screening of lung cancer, the detection rate and consultation rate of small lung nodules have gradually increased [7]. VATS lobectomy and mediastinal lymph node dissection is one of the most common operations applied during thoracic surgery [8]. However, it is necessary to construct a predictive model of the difficulty of the operation. The patients’ clinical data that are readily available before the operation and some noninvasive preoperative examinations can be used to predict the difficulty of the patient's upcoming operation. Knowing the predicted surgical difficulty in advance has the following benefits: (1) To help clinicians make surgical plans. A greater degree of difficulty suggests the possibility of conversion to thoracotomy, and increasing the number of incisions could reduce the difficulty of the surgery. (2) It is helpful for the education of clinicians to predict the difficulty of the operation so that inexperienced doctors can start with low-difficulty operations before moving on to more difficult ones. (3) The output of the nomogram is concise and comprehensive, which is conducive to the patient's understanding of their situation, and our predictive factors are based on the patient's routine clinical data, which will not place additional cost burdens on patients or subject them to additional invasive examinations. In this way, the doctor's ability to communicate with patients before the surgery will also be improved.

Previous studies have established predictive models for laparoscopic cholecystectomy [9], predictive models for liver resection [10], and models for radical resection of rectal cancer [11]. Ibrahim et al*.* [9] demonstrated that male gender (*p* = 0.023), age (*p* = 0.000), body mass index (BMI) (*p* = 0.000) and pre-operative endoscopic retrograde cholangiopancreatography (ERCP) (*p* = 0.001) are the variables associated with the level of the surgical difficulty of the laparoscopic cholecystectomy. Escal et al*.* [11] proposed the application of scoring system to predict surgical difficulty in patients with rectal cancer, which is related to BMI at least 30 kg/m^2^ (*P* = 0.021), coloanal anastomosis (versus colorectal) (*P* = 0.034), intertuberous distance less than 10.1 cm (*P* = 0.041) and mesorectal fat area exceeding 20.7 cm^2^ (*P* = 0.051). Since there is no objective standard to measure the difficulty of the operation, most of the previous studies have used the operation time, blood loss, postoperative complications, etc., to estimate the difficulty of the operation [12–15]. The intraoperative rehydration volume per unit weight was the outcome we initially sought to predict, but we were unable to access any precise values, the difficulty of data tracing, and the individual differences among the patients themselves made us finally choose the operation time as the predictive value.

Our results show that a history of lung disease (lung surgery history), accompanied by mediastinal lymph node enlargement or calcification, FEV1/FVC on the lung function test and FEV1 on the lung function test as a percentage of the predicted value would lead to a prolongation of the operation time to varying degrees. For the four related variables, we applied the variance inflation factor to detect the collinearity. The results are as follows: history of lung disease (vif = 1.01), mediastinal lymphadenopathy or calcification (vif = 1.09) FEV1/FVC on the lung function test (vif = 1.61) FEV1 as a percentage of the predicted value on the lung function test (vif = 1.63), when 0 < vif < 10, there is no collinearity among related variables, which indicated that the four related variables were not confounded by each other. Some studies have shown that age, gender and BMI have an impact on the duration of surgery, but the above factors were not significantly correlated in our study [9, 13]. Coagulation function indicators and other indicators have also been reported in previous studies, which will lead to an extension of the operation time, but in our study, we found the opposite. This may be because the operation in this study was elective surgery. In the perioperative preparation of the patients, the test indicators are adjusted to be acceptable for surgery, and smoking cessation education may be one reason for the failure of the smoking history to show a positive result [16]. A history of lung surgery or a history of lung disease may lead to pleural adhesions, lymph node calcification, and decreased lung compliance, which increase the difficulty of exposure to surgical vision and lymph node dissection. For the examination of lung function, only FEV1 accounted for the predicted value, along with FEV1/FVC, which is mainly related to lung compliance. Poor lung compliance is related to lung collapse during one-lung ventilation, which may increase the difficulty of exposure to intraoperative vision and thus affect the operation time. We believe that the degree of lung diffusion, which did not produce a positive result, is the reason why it is mainly related to the recovery of postoperative lung function and the frequency of postoperative complications. Preoperative chest CT to confirm whether the patient has enlarged or calcified lymph nodes can also provide a preliminary assessment of the difficulty of the operation because this may increase the difficulty of mediastinal lymph node dissection.

**Fig. 9:**
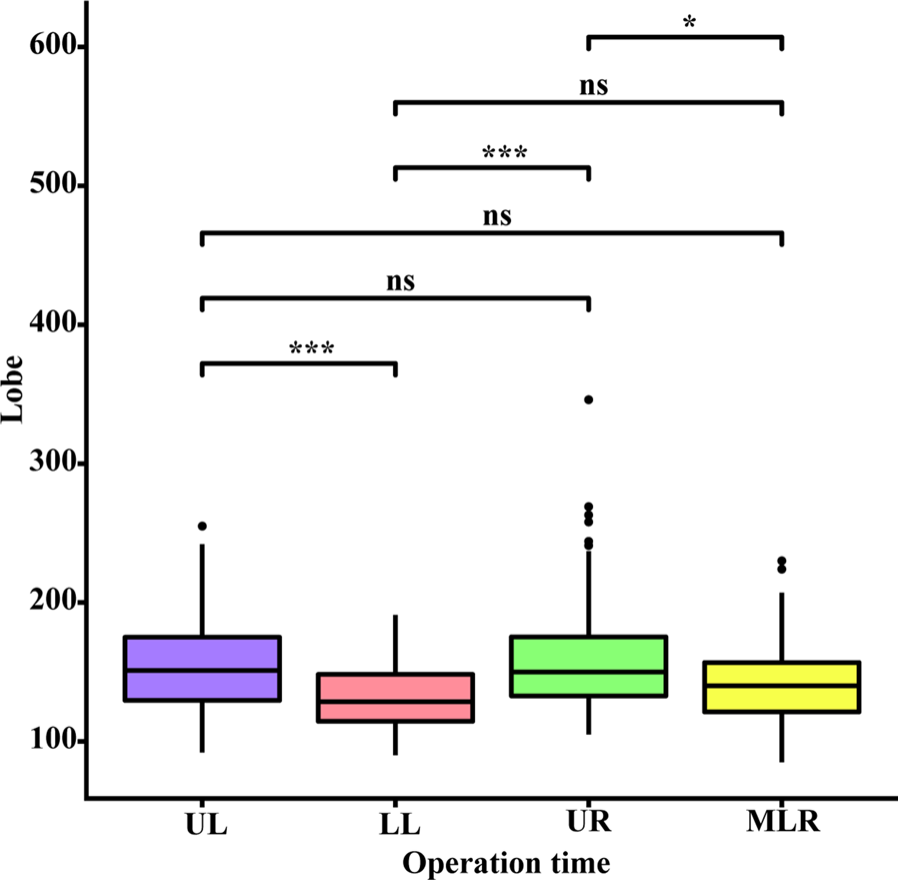
A box plot showed the difference in operation time between different lung lobes *UL* left upper lobe, *LL* left lower lobe, *UR* right upper lobe, *MLR* right middle lobe, right lower lobe

Due to the large number of preoperative variables included, we used Lasso screening in this study. In the end, we did not include fissure and lung lobe variables in the final model. As an influencing factor, lung fissure showed a correlation with the length of operation in the univariate logistics regression. However, in the two-way stepwise regression and Lasso regression, the lung fissure was not included in the final meaningful variable. According to the PROBAST score, it is a biased approach to use single-factor regression to screen first, and then multi-factor regression to build a model for the variables selected by single-factor regression. Therefore, we did not adopt this traditional model building method. We believe that for a complete model, it is appropriate to include all variables to be screened into the model from the very beginning [17]. We drew a box plot to show the degree of differentiation in the operation duration between different pulmonary lobes. The degree of distinction in the length of the operation is shown in Fig. 9. Since the surgeon we selected was an experienced thoracic surgeon, he failed to show a significant difference in the final time statistics when dealing with different lung lobes. However, based on clinical experience, we believe that different lung lobes have different surgical difficulties. We recommend that patients be grouped according to different lung lobes and then predicted by the model.

### Limitations of the study

The following points are unavoidable problems in our research. First, we chose the operation time to reflect the difficulty of the operation. In most cases, it can be considered that the longer the operation time, the greater the difficulty. It is difficult to find a gold standard for objectively judging the difficulty of an operation. Second, some of the factors that complicate surgeries are difficult to predict or know about in advance, such as pleural adhesions, portal lymph nodes that are difficult to completely remove, or bleeding due to intraoperative mishandling. Finally, as mentioned in above, some preoperative prediction variables are difficult to obtain. This study is more committed to some simple variables that are easy to obtain in the routine clinical diagnosis and treatment process. These variables acquired from the general information of patients or the results of non-invasive routine examination, therefore, the accuracy may be slightly insufficient compared with the results of invasive examination.

## Conclusion

Our retrospective study identified four preoperative variables that are correlated with a longer surgical time and can be presumed to reflect more difficult surgical procedures. Our prospective study verified that the variables in the predictive model (a history of lung disease, FEV1 accounting for the predicted value, FEV1/FVC, mediastinal lymph node enlargement or calcification) are related to the difficulty of the surgery. Based on these results, we recommend preoperative evaluation of patients undergoing VATS lobectomy and mediastinal lymph node dissection. These findings need to be validated in a sufficiently large prospective multicenter study. This model will become an important auxiliary tool for planning clinical programs.

## Data Availability

The datasets generated or analyzed during the current study are not publicly available due to the privacy of patients enrolled but are available from the corresponding author on reasonable request.

## Abbreviations

FEV1: Forced expiratory volume in the first second
FVC: Forced vital capacity
predFVC: Forced vital capacity in predicted
pred FEV1: Forced expiratory volume in the first second in predicted
OR: Odds ratio
CI: Confidence interval
AUC: Area under curve
ROC: Receiver operating characteristic
NCCN: National Comprehensive Cancer Network
VATS: Video-assisted thoracoscopic surgery
DCA: Decision curve analysis
BMI: Body mass index
